# An update on the use of cryoablation and immunotherapy for breast cancer

**DOI:** 10.3389/fimmu.2022.1026475

**Published:** 2022-10-27

**Authors:** Akindele Olagunju, Tia Forsman, Robert C. Ward

**Affiliations:** ^1^ Department of Diagnostic Imaging, Rhode Island Hospital, Women & Infants Hospital, The Warren Alpert Medical School of Brown University, Providence, RI, United States; ^2^ Department of Diagnostic Imaging, The Warren Alpert Medical School of Brown University, Providence, RI, United States

**Keywords:** cryoablation, immunotharapy, breast cancer, tumor specific antigen, synergistic, abscopal effect, tumor infiltrate lymphocyte, checkpoint inhibition blockade

## Abstract

The use of cryoablation, a minimally-invasive image-guided technique to target and kill cancer cells, continues to gain traction within the medical field and with patients. This includes the use of cryoablation for the treatment of small breast cancers and focal sites of metastatic disease. In comparison to open surgical approaches, length of hospital stay and recovery time are decreased with the use of cryoablation. Research studies have also found that cryoablation may actually enhance tumor susceptibility to immunotherapy agents. Immunotherapy enhances a person’s own immune system to identify and attack cancer cells. It is proposed that after cryoablation there is increased expression of tumor specific antigens which the body can recognize as foreign invaders and with the combination of immunotherapy, result in an even more robust and efficient attack on the cancer cells. In this review we aim to highlight some of the recent advances in cryoablation which support the potential for cryoablation to induce these tumor-specific immune responses and thus supporting the use of combining cryoablation and immunotherapy for the treatment of breast cancer.

## Introduction

Continued technological innovation and advancements in the medical field has made it possible for surgeries that were once thought to require an open approach to now have more minimally invasive options. One of these less invasive options is the use of cryoablation. Cryoablation is a minimally invasive radiology technique to percutaneously target abnormal tissue and freeze them to extremely low temperatures resulting in cytotoxic effects and destruction of intracellular contents (1). Cryoablation has been used in the treatment of multiple solid malignancies, including liver, lung and renal malignancies. The use of cryoablation for the treatment of small breast cancers and focal sites of metastatic disease has continued to gain traction with patients and providers as the procedure is minimally invasive and can be performed in an outpatient setting given the procedure requires only the use of local anesthetic. Even more revolutionary is the idea that cryoablation may lead to increased expression of tumor specific antigens which the body can recognize and use to better identify and eradicate cancer cells especially when combined with immunotherapy agents (1).

The goal of immunotherapy is to flag cancer cells as foreign so that they can be recognized by the patient’s own immune system (2). The most extensively studied immunotherapy agents for treatment of breast cancer are immune checkpoint blockade agents (3). Immune checkpoint blockade removes the brake on the activated immune system resulting in continued antigen-specific recognition and response (2). Overall, studies of these immunotherapy agents for breast cancer have shown limited efficacy, likely due to low mutational burden of the tumors and lack of T cell specific peptides presented by the tumor to the T cells (4). It is hypothesized that combining a technique which can produce increased expression of tumor specific antigens, such as cryoablation, may increase the efficacy of immunotherapy for treatment of breast cancer. These immune checkpoint inhibitors include cytotoxic T-lymphocyte antigen-4 (CTLA-4), programmed cell death 1 protein (PD-1) and programmed cell death ligand 1 (PD-L1). This review aims to provide an update on ongoing trials investigating these immunotherapy agents and if the results continue to support the combination of these agents with cryoablation to increase their efficacy.

## Regulation of the immune system

In order for the immune system to attack cancer cells, it needs to be able to recognize the cells as foreign. This occurs *via* recognition of aberrant antigenic protein or over expression of normally repressed genes by the tumor (2). To accomplish this, antigen presenting cells (APCs) must first recognize and bind the target, followed by endocytosing the target and degrading the target in order to display it on their major histocompatibility complex (MHC) for the T cells to recognize (5). After binding to the MHC of the APC, the T cell requires a second signal for activation through the B7 costimulatory molecule expressed by APCs which binds to CD28 on the T cell resulting in T cell clonal expansion.

A deactivation mechanism must also be in place to balance the activation of the immune system and prevent unregulated immune responses which could lead to autoimmunity. One way this is done is through cytotoxic T-lymphocyte associated protein 4 (CTLA-4) on the T cells binding to B7 on the APC instead of CD28, resulting in downregulation of T cell clonal expansion. The critical role that CTLA-4 plays in suppressing the immune system is what led to it being the first immune checkpoint receptor targeted for cancer immunotherapy (6). Another critical checkpoint mechanism to prevent harmful over activation of the immune system is the coinhibitory molecule, PD-1 found on the T cells, which binds to PD-L1. The binding of this molecule to its ligand results in inhibition of the immune response and promotes self tolerance.

## The importance of tumor-infiltrating lymphocytes

In regard to breast cancer, studies of immunotherapy response predictors have focused on tumor PD-L1 expression, tumor mutation load and tumor infiltrating lymphocytes (TILs) (2). TILs include T cells, macrophages, Natural killer (NK) cells and dendritic cells, which infiltrate tumor tissue in varying degrees. Studies have shown that the greater the amount of TILs in breast tumors, the greater the response to neoadjuvant chemotherapy and better overall survival rates (7). Breast cancers which demonstrate a significant amount of TILs are considered lymphocyte predominant breast cancers (LPBCs) and these tumors have the most favorable response to neoadjuvant chemotherapy. Not only is having a large amount of TILs within the tumor important, the composition of the TILs also plays an important role in immune response against the tumor. Studies have shown that dendritic cells, M1 macrophages, TH1 CD4+ T cells, cytotoxic CD8+ T cells and NK cells protect against tumor growth, while M2 macrophages, myeloid-derived suppressor cells (MDSCs), neutrophils, and certain regulatory T cells (Tregs) can promote tumor growth (8).

Tumor cells can use multiple mechanisms to escape recognition by the immune system. They can decrease the amount of TILs in the tumor microenvironment, prevent the activation of TILs or even induce factors to recruit tumor favoring TILs. It makes sense that an effective approach to the treatment of breast cancer is also a multifaceted treatment approach.

## Targeting checkpoint inhibition

One specific mechanism that cancer cells take advantage of to avoid the immune system is the upregulation of the amount of PD-L1 they express (9). PD-1/PD-L1 interaction functions in the effector phase of T cell activation primarily in the peripheral tissues (10). Once the T cell’s PD-1 interacts with the tumor’s PD-L1, this results in inhibited proliferation of the T-cell resulting in diminished host antitumor immune response (7, 10). CTLA-4 also has a similar inhibitory role by downregulating the priming phase of T cell response. T cells are primed in secondary lymphoid organs by establishing stable interactions with antigen presenting cells (11). Once a stable interaction is achieved, T cells can proceed onto clonal expansion and mount a robust immune response. But when CTLA-4 inhibitory mechanisms are active, T cells do not achieve a stable interaction with the APC and do not proceed to clonal expansion, or in the case of PD-1, already activated T cells are effectively turned off (10).

**Figure 1:**
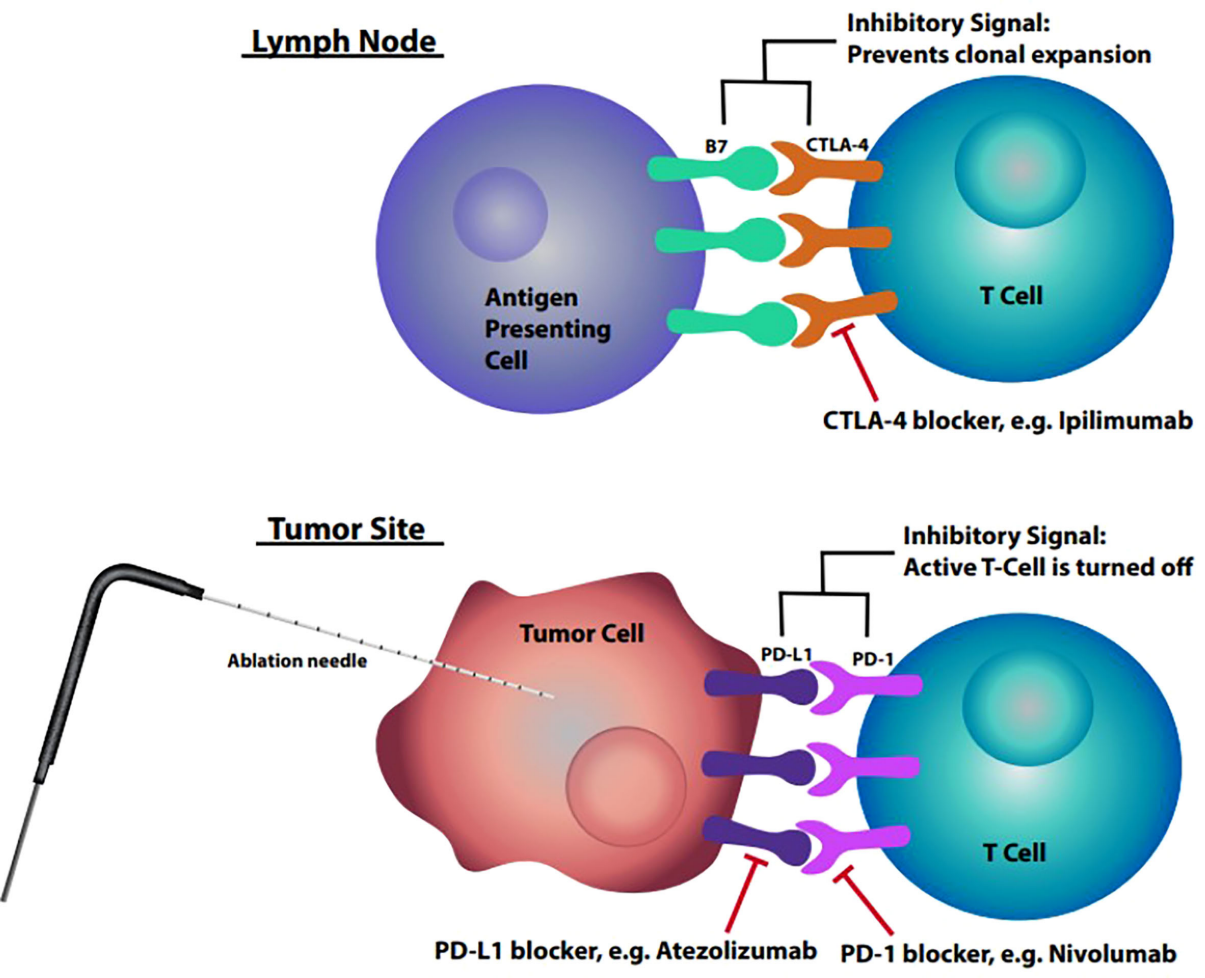
Lymph node: Interaction between B7 found on antigen presenting cell and CTLA-4 on T cells within the lymph nodes resulting in inhibitory signal preventing clonal expansion. Tumor site: Interaction between upregulated PD-L1 on tumor cells and PD-1 on T cells resulting in inhibitory signal deactivating the T cell.

Continued understanding of the roles that CTLA-4 and PD-1/PD-L1 play in the immune system has led to them becoming major targets for immunologic agents. One of these agents is Ipilimumab, a fully human monoclonal antibody which blocks CTLA-4 thus promoting antitumor immunity. Ipilimumab has been found to improve survival in patients with metastatic melanoma (12). The exact mechanism by which anti-CTLA-4 antibodies induce an antitumor response is unclear but research suggests that the blockage affects the priming phase by supporting the activation and proliferation of a higher number of effector T cells (10). On the other hand, blockade of PD-1 affects the effector phase, restoring immune function to T cells in the peripheral tissues that had been turned off. Blockades of CTLA-4 and PD-1 function at different times in the immune cascade and at different locations (**Figure 1**), leading to the hypothesis that these therapies may have an additive or synergistic effect in the treatment of cancer (10). One key limitation in the use of immune checkpoint inhibitors is the ability for the individual patient’s immune system to recognize and react to tumor specific antigens once the blockade of the inhibitory signals has been achieved.

## The role of cryoablation

**Figure 2:**
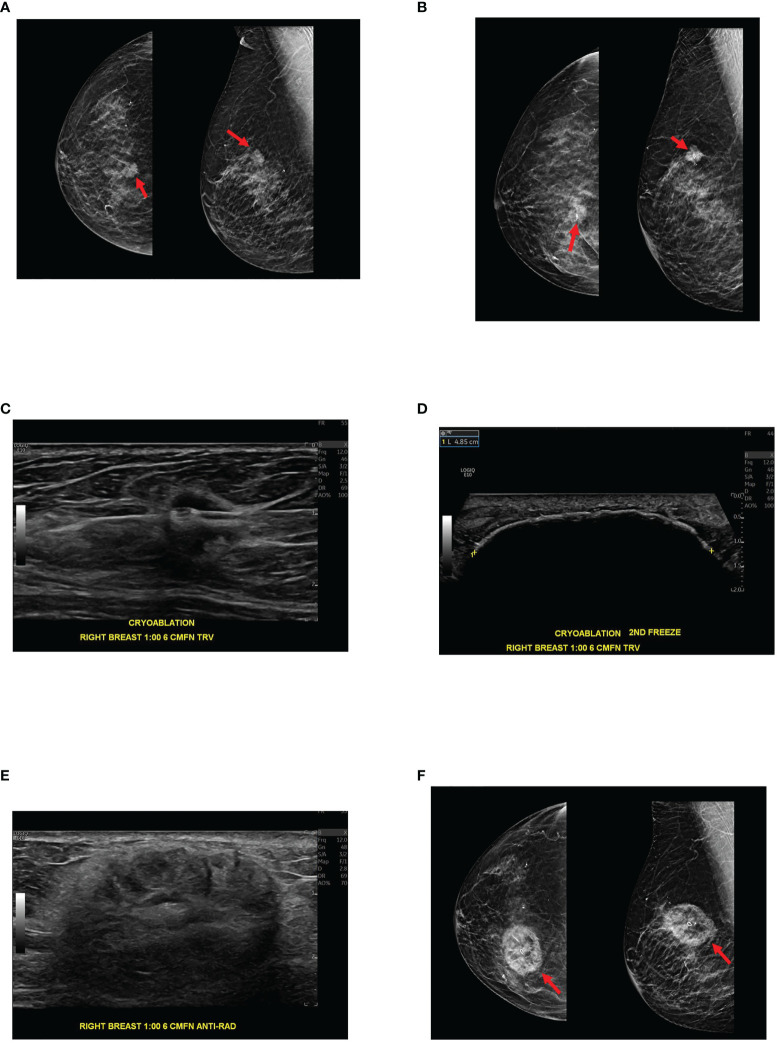
89-year-old female with biopsy-proven 1.3 cm right breast invasive ductal carcinoma, Nottingham histologic grade 2, ER/PR + HER2 -, presents for cryoablation. **(A)** Mammographic images with digital breast tomosynthesis CC (craniocaudal, left image) and MLO (mediolateral oblique, right image) projections demonstrate a suspicious high-density spiculated mass within the right breast at 1 o’clock, posterior depth. Ultrasound-guided biopsy was subsequently performed (not pictured). **(B)** Digital breast tomosynthesis CC (left image) and ML (mediolateral, right image) projections demonstrate the biopsy clip centered within the mass following ultrasound-guided tissue sampling. Pathology was consistent with invasive ductal carcinoma. **(C)** Ultrasound demonstrates cryoablation probe placement through the target lesion before initiation of the first freeze cycle. **(D)** Ultrasound shows the ice ball enveloping the target lesion and the surrounding tissues at the conclusion of the procedure, immediately after the second freeze cycle. Note the ice ball long axis measures 4.9 cm. **(E)** Three months after cryoablation, ultrasound demonstrates expected post cryoablation changes as a mixed echogenic mass surrounding the ablated tumor. **(F)** Three-month post cryoablation digital breast tomosynthesis CC and MLO projections demonstrate expected post cryoablation changes with the biopsy clip centered within the ablation zone, confirming appropriate cryoablation targeting.

Cryoablation is a minimally invasive image-guided procedure that is used to target abnormal tissue and freeze them to extremely low temperatures resulting in destruction of the involved cells (**Figure 2**). The use of cryoablation for treatment of fibroadenomas is well established with the procedure producing less pain, requiring less anesthesia and quicker restoration of normal to near-normal breast architecture than surgical excision (13). The use of cryoablation as an alternative to surgical resection of small early stage invasive ductal breast carcinoma is currently being investigated and to date results have been highly successful. The efficacy of cryoablation is based on the cytotoxic effects of cold that produce both instant and delayed destruction of cellular ultrastructure resulting in the release of intact tumor specific antigens (1). These intact tumor specific antigens can be recognized by the immune system and can activate the immune system to mount a response to the tumor (**Figure 1**).

To achieve this, cryoablation uses two freeze-thaw cycles. During the first freeze phase, not only is there damage due to the formation and growth of ice crystals, but water also freezes in the extracellular space faster than in the intracellular space resulting in an osmotic gradient that draws fluid out of the cell (1, 2). During the thaw phase, the osmotic gradient is reversed resulting in water rapidly entering the cell and causing cell rupture, releasing previously unattainable intact tumor antigens to the circulation for APCs to recognize (1, 2). The second cycle results in an enhanced and enlarged area of tumor destruction because tissues that were injured during the first freeze phase conduct cold temperatures even more efficiently (1). Cell death occurring by the cooling injury results in coagulative necrosis which mostly involves the central tissues of the ablation zone, while the tissues at the outer zone of the cryoablation zone are not destroyed by exposure to the cold temperature, but instead they undergo delayed apoptosis due to mitochondrial injury (14, 15). Although cell death through apoptosis is considered to be immunosuppressive, it is believed that there is a complex interplay between apoptosis and necrosis which is essential to determine the immune response to antigens (16).

This complex interplay between cell death *via* apoptosis and necrosis may be mediated by local inflammatory responses caused by cryoablation resulting in the release of proinflammatory cytokines such as IL-1, IL-6 and TNF-alpha which activate the immune system instead of the typical immunosuppressive response seen with apoptosis alone (14). In comparison to radiofrequency ablation, microwave ablation and radiation therapy, which induce cell death by heat, cryoablation induces a greater postablative immune response (14, 15). This is likely because these heat based techniques lead to protein denaturing and damaged antigens while cryoablation results in increased amounts of undamaged antigens that get released into the circulation which can induce tumor specific immune responses. Tumor specific immune responses have also been described in reference to radiation therapy where a phenomenon known as the abscopal effect is seen, in which following radiation of the primary tumor there is regression of distant metastatic lesions (14). The theory behind this effect is that after radiation treatment, tumor specific antigens can be released for the immune system to recognize and use to stimulate a tumor specific response which reflects a systemic link to local response (17). This phenomenon has also been hypothesized to occur with cryoablation, given its ability to cause the release of tumor specific antigens. In addition to the abscopal effect, studies have shown that cryoablation can affect the TILs in the distant tumor. In a study which aimed to analyze the effects of local cryoablation on distal tumor microenvironment in mouse models, it was found that there was an increase in the number of immune effector cells in the microenvironment of distant tumors and a decrease in the number of immunosuppressive Treg cells (18).

## Combined therapeutic approach

The ability for cryoablation to induce tumor specific immune responses and the ability for checkpoint inhibitors to remove the brakes from the immune system (**Figure 1**) has led to the belief that combining the two therapies could have a synergistic effect against tumors (2). Pilot studies have been performed to demonstrate this synergism and further evaluate the role cryoablation plays in the abscopal event. One preclinical study utilized a murine model of breast cancer to study TILs in distant tumors as a potential measure of the abscopal effect from cryoablation (17). In the study, two tumors were implanted at different sites into each mouse, where one tumor was targeted for local therapy (either surgical resection or cryoablation), followed by harvesting the other distant tumor one week later to study the systemic effect of the local treatment (17). The results of the study demonstrated increased TILs in the distant tumor with a significant increase in TILs in the cryoablation group. Another major finding from this study was that 40% of the mice in the surgical resection group developed both tumor recurrence and metastatic disease to the lung, and when those tumors were examined, they were the only tumors in the experiment that did not demonstrate increased TILs compared to the baseline control tumor. This finding points to the importance of TILs within the tumor microenvironment and that cryoablation results in a robust antitumor response both locally and systemically, thus demonstrating the abscopal effect (17).

The ability of cryoablation to create a robust antitumor response is why many studies hypothesize that its combination with immunologic agents would result in a synergistic effect against cancer cells. Studies examining the effects of antibodies against immune checkpoint molecules have only produced a modest response rate in breast cancer, which is believed to be due to a lack of preexisting recognition of the tumor by the immune system (19). This is why a more effective treatment approach would be the combination of checkpoint blockade with methods that cause increased tumor specific antigens for the immune system to recognize, which can be seen with cryoablation. In a pilot clinical study examining the safety of preoperative single dose ipilimumab (anti-CTLA-4) and/or cryoablation in 19 women with early-stage breast cancer, not only was the combination safe, favorable intratumoral and systemic immunologic effects were also noted (19). In the study, the group that received Ipilimumab and cryoablation demonstrated the highest expression of inducible costimulator (ICOS) which, in murine models, is part of the pathway for increased antitumor activity associated with anti-CTLA-4 agents. This group also demonstrated sustained proliferation of CD4 and CD8 cells. These findings help to support the potential synergy of combining cryoablation and immunologic agents.

This synergism between cryoablation and immunologic agents is presumably due to the role these treatment techniques play in *de novo* adaptive immune response. For an immunologic agent to achieve an optimal immune response to tumors, it should be able to facilitate four critical components of *de novo* adaptive immune response, including tumor antigen release, tumor antigen presentation, diminished immune suppression, and tumor antigen-specific T cell activation (20). Cryoablation is thought to play a role in tumor antigen release and presentation, while immunologic agents, like ipilimumab, play a role in diminished immune suppression and increased tumor antigen-specific T cell activation. In a pilot clinical trial involving 18 women with early-stage breast cancer who were treated preoperatively with cryoablation, single dose ipilimumab or cryoablation combined with ipilimumab, synergism was demonstrated by an increased amount and diversity of TILs. One of the aims of this study was to examine the effects of cryoablation and/or ipilimumab on intratumoral T cell density and T cell clonality. T cell clonality describes whether T cell-populations are oligo-clonal (reacting to one or a few antigens) or polyclonal (reacting to many different antigens) (20). The group that received only ipilimumab demonstrated increased T cell density. The group that received only cryoablation demonstrated decreased T cell density but an increased polyclonal shift. This latter finding is consistent with the idea that cryoablation leads to the death of both tumor and TILs in the tumor microenvironment while releasing a broader variety of tumor specific antigens for the immune system to recognize. When cryoablation and immune checkpoint inhibition were combined, there was a mixed effect on T cell density, but compared to monotherapy, there was a greater number of high-magnitude clonal expansion, suggesting that combination therapy mediates rapid proliferation of a small subset of T cell clones (20).

The findings of these studies are promising but limited by the use of animal models and by limited sample sizes of clinical studies. Multiple new ongoing clinical trials are now underway to further examine the synergism of cryoablation and immunotherapy. These include NCT02833233, NCT03546686 and NCT04249167 (ClinicalTrials.gov) (**Table 1**).

**Table 1:** Ongoing Clinical Trials of Combined Cryoablation with Immune Checkpoint Inhibitors for the Treatment of Breast Cancer.

Identifier	Phase	Study Design	Type of Immunotherapy	Primary Outcome Measure	Estimated Study Completion date
NCT02833233	N/A	Single group assignment, open label	Ipilimumab (anti-CTLA-4) Nivolumab (anti-PD-1)	Number of adverse events	June 2023
NCT03546686	Phase 2	Single group assignment, open label	Ipilimumab (anti-CTLA-4) Nivolumab (anti-PD-1)	Event-Free Survival	June 2024
NCT04249167	Early Phase 1	Single group assignment, open label	Atezolizumab (anti-PD-L1) Nab-Paclitaxel (anti-microtubule)	Safety and Feasibility	March 2025

N/A, Not Available.

NCT02833233 is a trial in progress in which the safety of combining Nivolumab (anti-PD-l), Ipilimumab (anti-CTLA-4) and cryoablation is being examined for women with early stage breast cancer. The primary outcome for this study is the number of adverse events. NCT03546686 is a phase II trial looking at the impact of pre-operative cryoablation, Ipilimumab and Nivolumab on 3 year event free survival in women with triple negative breast cancer after taxane-based neoadjuvant chemotherapy. NCT04249167 is a phase I trial study examining the side effects and feasibility of cryoablation, Atezolizumab (anti-PD-L1) and Nab-paclitaxel [solvent free, albumin bound nanoparticle formation of paclitaxel which promotes assembly of tubulin into microtubules and prevents their dissociation resulting in the blockage of cell cycle progression and inhibiting the growth of cancer cells (21)] in treating patients with triple negative breast cancer that has spread to nearby tissue or lymph nodes or has spread to other sites within the body. The results of these pending trials will greatly inform the discussion regarding the role of combination therapy in the treatment of breast cancer.

## Conclusions

Continued research in the field of cryoablation for the treatment of small breast cancers and the role cryoablation plays in the immune system is essential for continued development of effective breast cancer treatment regimens. Cryoablation is known to not only destroy target cells, but can also lead to the circulation of intact tumor specific antigens for the immune system to recognize and react to. With our current understanding of checkpoint inhibition and the effects of blocking PD-1/PD-L1 and CTLA-4, the potential for these agents to have a synergistic effect with cryoablation must be studied. Clinical trials examining this relationship are currently underway and their results will help evolve our understanding of how to treat not only localized small breast cancers but also nodal and distant metastatic disease.

## Author contributions

AO, TF and RW contributed to the writing, reading, editing and approval of the submitted version of this review article. AO specifically performed literature reviews to incorporate into the Review article. TF specifically designed the graphics. RW specifically oversaw the entire Review. All authors contributed to the article and approved the submitted version.

## Conflict of interest

The authors declare that the research was conducted in the absence of any commercial or financial relationships that could be construed as a potential conflict of interest.

## Publisher’s note

All claims expressed in this article are solely those of the authors and do not necessarily represent those of their affiliated organizations, or those of the publisher, the editors and the reviewers. Any product that may be evaluated in this article, or claim that may be made by its manufacturer, is not guaranteed or endorsed by the publisher.
